# Challenges and Limitation Analysis of an IoT-Dependent System for Deployment in Smart Healthcare Using Communication Standards Features

**DOI:** 10.3390/s23115155

**Published:** 2023-05-28

**Authors:** Shrikant Upadhyay, Mohit Kumar, Aditi Upadhyay, Sahil Verma, Kavita, Maninder Kaur, Ruba Abu Khurma, Pedro A. Castillo

**Affiliations:** 1 Department of Electronics & Communication Engineering, Cambridge Institute of Technology (CIT), Tatisilwai 835103, India; shri.kant.yay@gmail.com; 2 Department of IT, MIT Art, Design and Technology University, Pune 412201, India; mohit.kumar@mituniversity.edu.in; 3 Department of Electronics and Communication Engineering, School of Engineering, Jaipur National University, Jaipur 302017, India; sweetcaditi@gmail.com; 4 Department of Computer Science & Engineering, Uttranchal University, Dehradun 248007, India; sahilverma@ieee.org (S.V.); kavita@ieee.org (K.); 5 Department of Computer Science and Applications, Guru Gobind Singh College for Women, Chandigarh 160019, India; maninderkaur@ggscw.ac.in; 6 Computer Science Department, Faculty of Information Technology, Al-Ahliyya Amman University, Amman 19328, Jordan; r.khurma@ammanu.edu.jo; 7 Department of Computer Engineering, Automation and Robotics, ETSIIT, University of Granada, 18012 Granada, Spain

**Keywords:** healthcare, IoT, wearable sensors, communication standard, cloud, security

## Abstract

The use of IoT technology is rapidly increasing in healthcare development and smart healthcare system for fitness programs, monitoring, data analysis, etc. To improve the efficiency of monitoring, various studies have been conducted in this field to achieve improved precision. The architecture proposed herein is based on IoT integrated with a cloud system in which power absorption and accuracy are major concerns. We discuss and analyze development in this domain to improve the performance of IoT systems related to health care. Standards of communication for IoT data transmission and reception can help to understand the exact power absorption in different devices to achieve improved performance for healthcare development. We also systematically analyze the use of IoT in healthcare systems using cloud features, as well as the performance and limitations of IoT in this field. Furthermore, we discuss the design of an IoT system for efficient monitoring of various healthcare issues in elderly people and limitations of an existing system in terms of resources, power absorption and security when implemented in different devices as per requirements. Blood pressure and heartbeat monitoring in pregnant women are examples of high-intensity applications of NB-IoT (narrowband IoT), technology that supports widespread communication with a very low data cost and minimum processing complexity and battery lifespan. This article also focuses on analysis of the performance of narrowband IoT in terms of delay and throughput using single- and multinode approaches. We performed analysis using the message queuing telemetry transport protocol (MQTTP), which was found to be efficient compared to the limited application protocol (LAP) in sending information from sensors.

## 1. Introduction

Smart health care and smart hospitals are concepts that have emerged as a result of innovation in the healthcare sector with the key supporting of technologies, especially the Internet of Things (IoT), individualized facilities and intelligent devoted machines (in terms of artificial intelligence). IoT is a rapidly evolving technology with potential for distributed computing and rapid information sharing in large-scale networks.

Since the COVID-19 pandemic [1], considerable investments have been made in health care, which an important sector in everyday human life. The increasing population and the rise in chronic diseases have increased the demand for hospital resources and monitoring technology [2]. Appropriate solutions and facilities are required to minimize the pressure on healthcare systems and severe patients [3]. A potential solution to minimize the load on healthcare systems is the use of IoT technology. Therefore, efforts have been devoted [4,5,6,7,8] to the development of innovative healthcare solutions. A large amount of research has been conducted on patient monitoring in specific diseases such as Parkinson’s disease and diabetes [7].

Many studies have focused on specific tasks, such as the combination of rehabilitation with regular tracking of patient progress [8]. The importance of emergency healthcare support has also been recognized [9,10] but has not been widely researched. Various studies have reviewed specific health-related technologies. For example, the authors of [11] conducted a review with a primarily focus on a commercially obtainable solutions, remaining problems and best possible applications. Various topics were considered in [12,13], including system storage, analysis and data mining, as well as system integration. The authors of [10] investigated big data control and sensing in terms of network-supported communication.

This paper represents a crucial contribution in that we explore all key aspects of end-to-end IoT related to health care and suggest a collective model that can be applied to any healthcare system based on IoT. This work is important because no such system for end-to-end communication and remote tracking of health issues has been established to date. Herein, we provide a detailed review of IoT technologies that related to the suggested model. The novelty of this article lies in its focus on aspects of IoT related to healthcare systems. Low-power networks are among the major needs in IoT systems. Herein, we compare standard licensed bands such as low-power Bluetooth, narrowband IoT, etc., with alternatives with specific attentiveness and discuss their suitability for different healthcare applications. Smartphones play an important role in daily life; these devices can be connected to various types of sensors to track the health status of individuals [14]. Such devices can sense and record various types of data for automatic and efficient control of health care [15]. IoT healthcare systems can also be effectively used to track and monitor human resources [16]. Cloud resources can be used to control healthcare data of healthcare through various strategies, such as data integration, storage flexibility and parallel processing, although all these methods are subject security issues [17]. Implanted sensors or wearable devices can be used for IoT healthcare applications using a limited power supply. However, frequent charging may be required, and the use of such technology may result in patient fatigue, requiring additional care support and influencing user involvement [18]. Cloud data centers consume considerable energy resources, increasing costs; on the other hand, cloud service requires minimal energy and provides low latency [19]. Security is one of the major concerns associated with healthcare monitoring, as crucial data can easily be corrupted by hackers.

**Figure 1:**
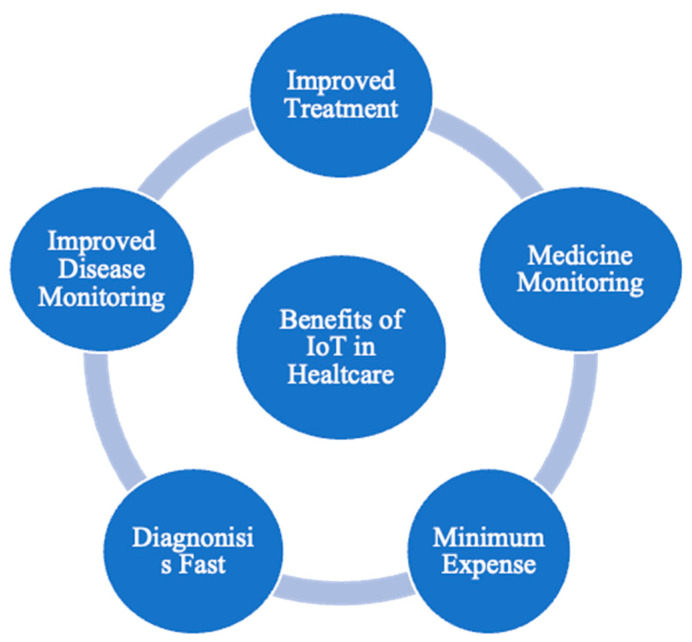
Advantages of IoT-dependent health care.

Therefore, data privacy and protection are important considerations in IoT healthcare systems with respect to data transmission [20]. Few researchers have proposed secure platforms or resources for healthcare systems based on IoT data transmission [21,22]. Therefore, in this study, we analyze a healthcare system in terms of computational duration, accuracy and development issues with the aim of arriving at a superior solution. The benefits of a smart healthcare system using IoT are shown in Figure 1.

## 2. IoT Challenges in Health Care

IoT has been deployed in various kinds of applications, supporting numerous healthcare services, including patient observation and smart homes for patients suffering from diabetes, mental illness, etc. Serious issues that arise in the healthcare systems are addressed below:

1. Information that is transferred from sensors to control devices and forwarded to invigilator centers affects the performance of data due to external noise. Architecture with improved construction can help to deliver data without disturbing the original pattern. A noise-supporting approach can also be adopted to optimize the strength of the data signal;
2. Various survival tracking techniques using ECG involve analysis of the acquired signal in a governed manner, which increases costs and results in error in the identification of the correct signal. Applying a machine learning approach for signal inspection can help to enhance performance and reduce costs;
3. As the quantity of sensors increases due to their widespread application and attached devices require greater energy for processing, chances of energy absorption and power leakage are increased. Therefore, an optimized methodology is required to minimize the utilization of energy;
4. Medical professionals require large amounts of data for clinical practice; the management of such data is quite challenging, and such issues should be efficiently addressed to minimize data loss;
5. Real-time communication is difficult in laboratories or clinics in which medical teams require immediate information from cardiologists, diabetologists, etc.;
6. The provision of in- and out-patient monitoring with available resources and facilities associated with different hospital is another challenge;
7. In order to provide adequate health care for every person, the availability of e-healthcare facilities should be increased.

## 3. Related Work

The distributed nature of the cloud environment makes it the best choice to store health-related data and provides a flexible means of remote patient monitoring by various experts. Therefore, the combination of cloud and IoT technologies is promising for the real-time processing of signals, which generally increases the complexity of the architecture required to receive and send information. In order to minimize the complexity between cloud and IoT technology, a framework is suggested to handle real-time IoT statistics, with unrelated scientifically based information verified in the cloud environment using tools such as hybrid services [23].

A framework based on service management for IoT devices is suggested for the cloud environment that consists of three types of layers and five crucial elements to serve consumers and gather information from patients, smart homes, smart healthcare devices, social networks, etc. The next level includes a service provider that provides physical assets, visualization, supervision service and security and privacy features. The last layer is the intermediate layer, which will help to manage services between consumers and providers, relying on available assets.

The authors of [24] suggested an IoT machine learning architecture for prior detection of human heart disease based on three-tier frameworks to gather data from sensors embedded in wearable devices. The suggested architecture is executed using Mahat (Apache) and Hbase (Apache) for data prognosis and cloud storage to help identify heart illness in the early phase.

The authors of [25] designed a framework to track patients suffering from arthritis and identify them in early stages. The proposed framework is based on three stages, the first of which involves gathering data from sensors, followed by information storage in the cloud and optimization of the collected data containing patient information such as uric acid levels and data on swelling in the body. Open stack Apache red displacement was used to design the model.

**Figure 2:**
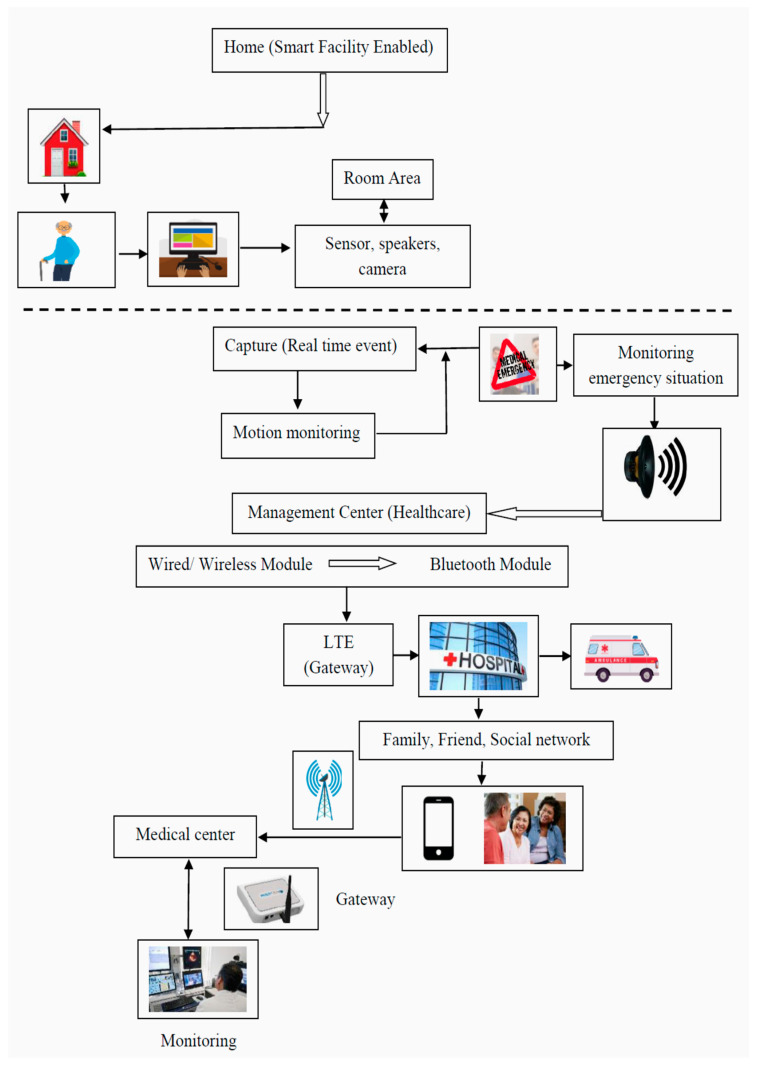
IoT framework for healthcare systems.

The authors of [26] designed and visualized sensor devices for household applications such as smart rooms for patients suffering from chronic diseases. No real-time data were considered in the experiment, as the proposed approach is prohibitively expensive. The framework of this approach can be assumed, and sensors can be used in place of cameras to minimize the total cost of the experiment. The outline of the proposed IoT framework is depicted in Figure 2.

The authors of [27] suggested a fuzzy ant tree classifier comprising a combination of of ant colony development, fuzzy regulation and a settlement tree, which was tested for an application involving organization of medical history. A thorough review of medical history can help to address issues that arise in elderly patients, supporting diagnosis and decision making with respect to pain management, medication, etc. The role of IoT in the proposed framework is to gather real-time information from sensors and identify health issues by verifying behavioral trends and physical need at home. Few researchers have conducted studies involving the support of elderly people using IoT tracking devices. Services for elderly people are often based on social platforms, enabling tracking using IoT devices. Sudden falls in elderly patients due to muscle pain can lead to death in some cases. To overcome such issues, an algorithm known as the fall identification algorithm was suggested to identify their condition and the specified area where the maximum chances of accident arise.

Open issues include cloud integration, big data handling, security, etc. Data received by IoT sensors can be effectively stored in cloud servers, and IoT devices can be used to transmit data to different cloud subsystems for disease identification. Cloud and big data integration may solve problems associated with medical data management. It is very important to analyze the limitations of data privacy in term of security to increase network trust.

The specified location contains RFID and information recognition data that can be used to easily track and minimize such cases. Such research can support elderly people by providing technology to enable safe homes where they can reside safely, effectively and, most importantly, comfortably in a monitored environment with the ability to continuously alert healthcare units and their family members in the event of alert signals [28,29,30].

Many researchers have suggested health-related devices to help society that may prove beneficial for the development of a smart healthcare system. Various types of pulse sensors have been developed for different applications. A chest-worn platform based on a wrist sensor is considered one of the most comfortable systems for the long term [31]. Photoplethysmography (PPG) sensors function using LED relay lights inside arteries to measure pulse variation and the rate and flow of blood oxygen in a form of a small wearable wrist sensor [32].

**Figure 3:**
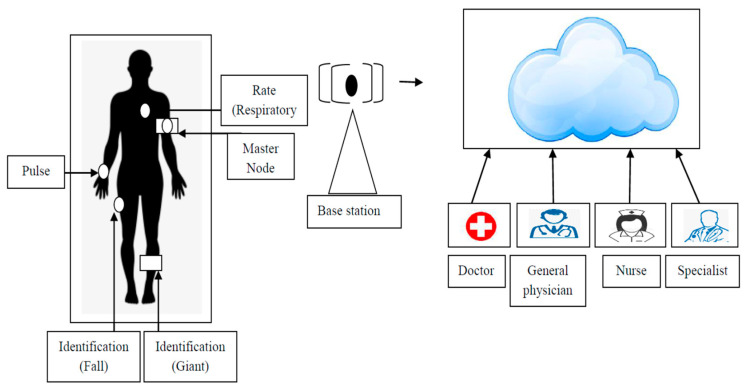
Proposed IoT-dependent model for healthcare systems.

Pressure sensors also support health care by manually measuring radial pulse using fingers, as depicted in Figure 3; the sensor is placed close against the wrist, and the pulse is measured to achieve a waveform. The authors of [33] proposed and verified a pressure sensor that is highly sensitive and flexible for the detection of pulse, which was found to provide satisfactory results. One drawback of the proposed sensor is that with increased sensitivity, noise is also increased due to the motion of the wearer. The use of an RF array based on an eccentric pulse sensor has also been proposed to measure pulse at various locations on the wrist, although noise is an issue due to motion [34].

Respiratory rate and breath count per minute can also be used in healthcare applications to identify various respiratory-related issues, such as lung cancer, panic attacks, tuberculosis, asthma, etc. The importance of respiratory rate in the field of health care has led to the development of many sensors, such as a nasal sensor relying on a thermistor [35].

The authors of [36] developed an optical base sensor that is sufficiently sensitive to measure vibrations arising from respiration. The proposed sensor was only verified in one test but was found to be feasible for all conditions in further tests. The authors of [37] developed a pressure sensor based on two plates containing capacitive and time-of-breathing plates that expand and contract during exhalation and inhalation, respectively. The authors reported a confidence of 94–95% in respiratory rate calculations compared to a nasal sensor. The authors of [38,39,40] suggested the use of a stretch sensor to measure respiratory rate based on tensile force and stretching during inhalation.

The authors of [38] introduced a sensor based on a ferroelectric polymer that generates a charge when tensile pressure is applied, acting as a transducer. The authors of [41] suggested a body temperature sensor based on a positive coefficient of temperature, the most common of which a negative coefficient or thermistor sensor.

The authors of [42] suggested that temperature can be accurately detected with using sensor technology embedded in textiles. However, wearable sensors designed for continuous and non-spreading blood pressure (BP) detection remain a challenge in the domain of health care. A number of attempts have been made to achieve accurate estimation of BP using pulse train duration [43,44,45,46], which is the duration of pulse between the heart and other areas, such as radial arteries or earlobes. The authors of [47] attempted to measure this property between the wrist and ear, whereas the authors of [48] attempted to calculate pulse train duration between the fingertips and palm. A sensor measuring the level of oxygen was implemented using pulse oximetry sensors. The authors of [49] suggested a low-energy oximeter for pulse detection with the aim of enhancing wearability. Two approaches can be used to minimize power absorption, the first of which is lower SNR (signal-to-noise-ratio) monitoring, which regularly calculates the SNR and regulates the stretch of duration, causing an LED to be in the “ON” condition. With higher SNR values, the LED reflects accurate readings. The second approach is phase-locked loop tracking, which calculates the probability of troughs and peaks of a photoplethysmographic signal, providing crucial real-time information.

The authors of [50] designed a pulse oximeter based on ear reflection, which was designed to identify the level of blood oxygen even when a patient is suffering from hypothermia, shock, fear etc., which may cause blood systematization, increasing the chances of an unidentified pulse rate in the fingertips. Reasonable precision was achieved in surgical patients; however, the authors concluded that these sensors can be used in combination with wearable pulse oximeters on the fingers but not as an alternative.

The authors of [51] suggested an oximeter that can be worn on the wrist that is concave in structure, avoiding external light, with improved noise resistance. The proposed device can also measure skin temperature in combination with three crucial sensors in a single wearable node. Many other wearable sensors have been proposed with a focus on tracking the specific condition of patients. ECGs (echocardiograms) are among the most common methods to measure heart-related issues, and various wearable devices have been developed to gather these signals. The authors of [52] suggested a helmet with an electroencephalogram (EEG)-enabled sensor feature to measure the activity of the brain to identify conditions such as sleep disorders, seizures and progress after head injury.

The authors of [53,54] suggested an EEG system for a specific purpose such as driver drowsiness identification or stress management in the form of a wearable headband EEG system to measure activity. The authors of [52] suggested an ECG sensor based on an armband to provide measurements with reasonable precision.

The authors of [55] discussed a triaxial accelerometer embedded inside a cellphone (smartphone) using a machine learning approach to detect the posture of the user using a suitable algorithm, achieving an accuracy of 90%. The classification approach for the detection of posture was found to be less accurate for fall detection [56], with an accuracy of 89% in an outdoor environment and 93% in an indoor environment. In [57], a previously proposed camera-based wearable system was combined with an accelerometer, achieving 91% precision in fall identification.

In [58,59] a gyroscope, accelerometer and magnetometer were used to accurately identify falls also; the authors also added a barometer for accurate identification of falls based on changes in height. In further work, accuracies of 98 to 100% were achieved in fall detection, and the authors suggested the immediate implementation of the proposed system in healthcare settings. For elderly patients, the gait identification method is quite useful to obtain knowledge of specific conditions such as Parkinson’s disease. Gait identification was considered in patients with Parkinson’s disease and those healing from a stroke in [60] using a wearable foot sensor designed to measure various parameters, such as walking speed, step length, etc.

In [61], a sensor developed to control prosthetics of the lower limb was used for gait detection in lower-limb amputees. The authors of [62] suggested placing three accelerometers on the ankle, knee and hip of Parkinson’s patients. A feature extraction approach was used in combination with an anomaly detection approach for gait freeze and anomaly identification in [63] using waist-worn embedded devices with microcontrollers and accelerometers (triaxial) to track gait. The authors of [64] suggested an optical sensor to measure blood glucose in the fingers, and in [65] a microwave ultra-band approach was used to measure the level of blood glucose through an earlobe sensor. The abovementioned is not only a special-purpose sensor for disease applications but should be considered in the design of any healthcare system to tracking any ailment.

## 4. Internet of Things and Health Care

IoT is still a somewhat new area of research and its utilization in health care remains a nascent field. Various developments have been in the use of IoT systems for health care. A standardized and common model for the upcoming head-to-foot IoT healthcare structure is suggested to guide the evolution of such a system. Various explanations of IoT exist; at the most basic level, it can be described as a network of individual devices inter-related via machine-to-machine transmission, allowing for the collection and interchanging of information [66,67,68]. Such mechanics can be applied in a wide range of industries, enabling the collection of large amounts of data. As a pilot technology of the Fourth Industrial Revolution [69] IoT mechanics have already been fruitful in many areas, such as smart stationing [70], agricultural fidelity [71] and management of water usage [72]. Remote health monitoring can be implemented for patients at home instead of in a hospital, minimizing pressure on hospital assets such as beds and doctors. Such an application could be beneficial for patients in rural areas, improving access to health care, as for the elderly living independently.

IoT technology can also improve access to healthcare resources by reducing pressure on systems and providing better facilities and constant monitoring. Remote health monitoring is subject to some limitations, the most important of which are related to security due to the massive quantity of sensitive data recorded in individual databases on a regular basis. Therefore, sensors must be recalibrated regularly to ensure that they are functioning correctly and to minimize service disruptions in the event that a patient is found to be outside of the coverage area of a base station or his/her battery is drained. Fortunately, such issues can easily solved, and steps can be taken to minimize such disadvantages. Remote health monitoring systems based on IoT are expected to become viable for healthcare settings in the near future. Many researchers have recognized the strengths of IoT as a suitable solution for remote health monitoring systems.

Many healthcare systems based on IoT are designed for a specific task, including diabetes supervision, rehabilitation, supportive surrounding living for the elderly, etc. Such systems have been structured for various applications and are strongly associated with enabling technologies. Several researchers are interested in the topic of rehabilitation and physical injury. For example, IoT technology can be used to develop a rehabilitation structure for individuals associated with their symptoms based on correlations with symptoms, disorders and therapies in a database.

Such a system requires the manual support of the doctor, who inputs symptoms and implements the suggested treatment in 90% of cases. If the doctor is completely satisfied with the system’s performance, no changes are required in the suggested plan of treatment [66]. The proposed model for a healthcare system using IoT is shown in Figure 3 and can be considered as a future system to guide decision making, serving as a strong foundation for various cases.

**Figure 4:**
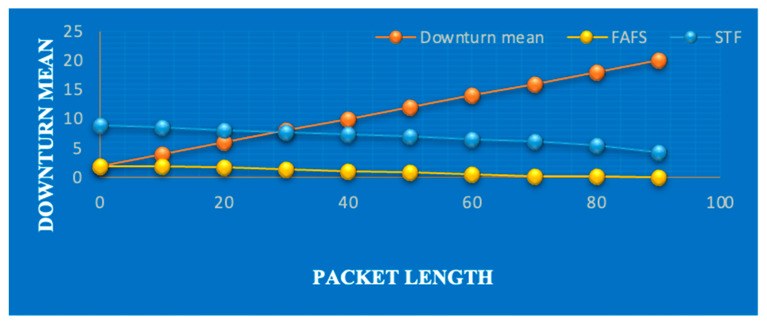
Comparison of scheduling approaches. STF denotes the small-task-first approach, and FAFS denotes the first-arrived, first served approach.

Various scheduling mechanisms have also been analyzed in recent years, showing that the scheduling mechanism performs well compared to the first-arrived and lower jump approaches in term of packet loss. Figure 4 shows previous attempts at scheduling mechanisms, with efficient performance of that priority-based approach. Therefore, it is very necessary to analyze the packet status, which is discussed in the Results section.

## 5. Long–Short-Range Communication

Standards used for communication in IoT healthcare applications are divided into two categories, i.e., long-range and short-range standards. Communication occurs among devices within a wireless area network, which are, in turn, connected to a base station and the central node of the WBAN. In this article, we consider both short- and long-range communication.

### 5.1. Long-Range Commmunication

Low-power networks (LPWANs) are a subgroup of long-range transmission standards with considerable applicability in IoT applications. The coverage area of LPWANs is several kilometers in urban areas, which is considerably longer than the traditional range of IoT communication technologies such as Bluetooth or Wi-Fi, the range of which is only a few meters. Therefore, a costly or nearby network is required for reasonable applicability in health care.

LBWANs offer advantages over cellular networks such as 3G, which are constructed for transmission of small amounts of data and are rarely suitable for the massive quantity of data required for healthcare purposes, including for general health tracking, receiving regular updates, emergency calls, etc., as well as rehabilitation applications for which monitoring updates may be necessary on a daily basis [73].

The principle of construction is also suggested for low-energy healthcare devices in order to ensure their function for a long duration before human intervention is necessary to change or recharge batteries.

This can reduce the patient risk of being disconnected, in addition to increasing convenience for the wearer. With such advantages, a low-power network is best-suited for the transmission of data from the master node to the cloud for data processing and storage. Lora WAN and Sigfox were found to be the most promising standards for low-power networks or LPWAN. Such standards are well-established and are suitable for upcoming standards including narrowband IoT [74].

In terms of suitability, the existing standard LPWAN was found to be efficient for IoT-based healthcare systems, which are listed and compared in Table 1.

#### 5.1.1. Sigfox

The most widely used and simplest low-power network standard is Sigfox with limited functionality. Sigfox is a protocol developed for the OSI model using the initial four layers. Its base station is similar to that in cellular networks; the antenna is mounted on a pillar based on a star topology network constructed in such a way that at the time of uplink, its battery efficiency is improved. It is also possible for a node to include a downlink, but it requires a request to receive the acknowledgement and is limited to four requests a day. The maximum range of Sigfox is around 9.5 km in urban environments using a suitable modulation scheme with a bit rate of 100 bps, which is quite low [75].

Its range can be further extended up to 50 km [76] but is limited in terms of high latency for healthcare applications. It operates in various unlicensed bands in different countries in Europe, working at 868 MHz, and 915 MHz in the US. No global band exists for the use of Sigfox, whereas some other low-power network technologies function at sub-GHz frequencies. It requires high bandwidth because it functions in the unlicensed band, which increases interference that may harm the healthcare system. In order to minimize this disadvantage, Sigfox follows three successive frames with a unique pseudo-random carrier using dissimilar propagation paths, which affect and improve the likelihood of an intact message being received, thereby reducing the chances of interference. Sigfox can handle approx. fifty thousand nodes using a single gateway to increase its network capacity. Compared to narrowband IoT, which covers around fifty thousand nodes, Sigfox supports 40 devices per household with an intersite length of 1732 m [77].

#### 5.1.2. LoRaWAN and LoRa

The basic and technical information for these two standards is mentioned in by the LoRa alliance. LoRa is a protocol function in the physical layer that uses a chirp spread spectrum approach with a wide bandwidth of nearly 125 kHz, providing long-range transmission, low power and good flexibility to environmental interference. LoRa WAN is constructed using the LoRa standard, and it follows a star topology network with asynchronous nodes and required communication when necessary. It uses scheduled messages from nodes that are suitable for long-term tracking applications, whereas event-based messages are used for emergency tracking. The high network capacity feature of LoRa WAN allows multiple messages to pass through forty thousand nodes. It has been implemented in various parts of Asia–Pacific, Europe and America, and with a high link budget of 155 dBm, information can transfer in a range of up to 5.5 kbps, which is considerably faster than Sigfox; this trade-off is valuable, as it enables a short delay that is essential for healthcare applications. It also operates on the unlicensed band at 915 MHz in the US and 868 MHz in Europe, offering advantages in terms of broad-spectrum accessibility. LoRa WAN is suitable for healthcare applications because of its network capacity, range and latency. Interference may be an issue, as it operates in an unlicensed band.

#### 5.1.3. Narrowband IoT

Narrowband IoT technology functions in a GSM-licensed band or LTE and covers a long range with low power required for communication. It relies on LTE (long-term evolution), and its hardware component and is used for effective and rapid deployment [78]. Narrowband IoT can be implemented in different ways and can coexist with available networks. The implantation node is available in standalone, in-band and guard-band forms.

In standalone mode, carriers of GSM are reframed and deployed for narrowband IoT, entering a newly available bandwidth. In the case of the guard band, it follows the bandwidth of the LTE carrier existing in the guard band. Finally, in the in-band mode, it reserves the physical resource of LTE [79,80]. One significant advantage of narrowband IoT is that it functions inside the licensed band and has less risk of interference. Its disadvantages are the higher cost of narrowband IoT compared to unlicensed standards. Devices require a connection fee to use LTE, but a decrease in interference makes this technology suitable for use in healthcare systems as QoS. It achieves a high sensitivity of about 164 dB, which covers a long range of up to 15 km with an uplink data rate of 250 kbps [81]. Because of the wide range and significant data rate, it is suitable for healthcare applications. The lifetime of battery functions at 5 wh was found to be 2.3 years in standalone devices, and a 50-byte message can be sent every 2 h in in-band mode, increasing the life of the battery up to 18 years.

Various applications depend on long-term tracking, which requires multiple transmissions per day, whereas emergency tracking systems send short “heart beat” information occasionally. In emergency scenarios, longer messages are required to track the exact status of a patient in real time. Narrowband IoT consumes less energy and therefore requires less interaction by the wearer, supporting a minimum of 52 of 548 nodes in a base station and 40 devices per home, which corresponds to a density equal to that of London, where every person could wear fifteen sensors and communicate successfully with the closest base station. Many other low-power standards have been developed for unlicensed and sub-GHz bands; however, fewer such standards have been deployed compared LoRa WAN and Sigfox. They consist of unique hardware features making them suitable for deployment on wide scales, including weightless, symphony link, narrow band-Fi and nWave.

### 5.2. Short-Range Communication

In healthcare systems, short-range technology is used in wearable devices for short-range communication among nodes, especially between the master node and sensor nodes, where data processing occurs. Short-range standards are used for different applications such as smart lighting in mesh networks. Here, we discuss the development of a small wideband network consisting of only a central node and a few sensors. Many short-range standards are available, the most well-known of which for IoT applications are Zigbee and low-power Bluetooth. The features of some standards used in short-range communication are listed in Table 2.

#### 5.2.1. Low-Energy-Based Bluetooth

Low-energy-based Bluetooth technology was developed by a special interest group to supply efficient energy standards that can be adopted by battery-based (coin-cell) wearable devices. The main objective of this technology is to activate IoT, connecting small interfacing devices to processing devices such as smartphones. Low-energy Bluetooth can be implemented using a star topology, which is quite useful for healthcare purposes. The master node behaves as the master of said topology, with the sensors attached. The sensors do not require direct communication with each other. The range of this technology is around 150 m in open conditions [82], with a shorter range in non-ideal scenarios.

This technology has quite a low delay of 3 ms and a data rate capacity of 1 Mbps [83], making it suitable for use in wideband healthcare systems in which nodes are physically proximal and extremely low delay is ideal in the case of emergency health situations. It operates in the 2.4 GHz band, which is also suitable for classical Zigbee and Bluetooth. This band sometimes generates some noise, but with the help of frequency hopping in channel selection and redundancy checking, the interface can be minimized to maintain its robustness, making it suitable for healthcare systems [84]. Power consumption is extremely low, at about 180 mAH, and the system can run for 18 h continuously. If the sensor sends information 2880 times, i.e., every 30 sec, then the battery can function for around 18–20 years. The use of a low-power program and careful hardware construction make it suitable for different healthcare purposes.

#### 5.2.2. Zigbee

Zigbee Alliance company introduced Zigbee standards to support low-energy, low-cost mobile-to-mobile communications. It is constructed using IEEE physical standard 802.15.4 [85], which is common to mesh networks but can also be used in a star network consisting of a wireless body network with one master node and various recognizing nodes. Various modules of Zigbee are available with different characteristics concerning its network capability, energy consumption and data rate.

Standard Zigbee has a range of 30 m in urban environment with 1 mW of energy for transmission [86]. A higher range of 90 m is available in Zigbee Pro in the same scenario with an output power of 63 mW, and Pro-900 can cover up to 610 m in urban environment scenario with a power of 250 mW [87]. Healthcare wireless body networks require 1 mW of power and short range for on-body exchange. The rate of data is 250 kbps for Zigbee Pro and Zigbee, whereas Zigbee Pro 900 has the highest data rate of 10 kbps. Subsequent modules have lower data rates depending upon power efficiency to cover a longer range. In the case of healthcare, it would be better to choose a higher data rate in order to minimize the delay in the system and ensure that critical health data are delivered on time.

Depending upon the module, Zigbee can operates in frequency ranges of 2.4 GHz, 900 MHz and 868 MHz, each of which are subject to interference issues. Wi-Fi and Bluetooth the 2.4GHz band, whereas various long-range communication systems operate in unlicensed bands. Collison avoidance using multiple carrier sense approaches is implemented to minimize collisions and retransmit messages [88]. Zigbee technology has been deployed in various health-related applications. An Alzheimer’s patient detection application with alerts was developed using a mesh network (Zigbee) [89]. Using Zigbee, a wearable ECG was developed to communicate with master tracking devices [90]. In biomedical applications, improved Zigbee was considered [91] using a low-power transmitter and receiver to increase reliability in terms of interference. Overall, Zigbee performs reasonably and is suitable for healthcare applications owing to its high level of security and robustness to interference.

## 6. IoT Security

Security is one of the major concerns for IoT because attackers or hackers can silently access data from sensors [92,93]. Therefore, it is crucial to know the various recent security approaches for IoT. One such approach known as IDP (IoT-dependent data placement) is used for privacy preservation. In this suggested approach, the main objective is to optimize the access of data concerning time and resource utilization and minimize power absorption according to the limitations of data safety [94].

Power minimization and privacy preservation can be achieved using NSGA-ii (non-dominated sorting genetic algo.) algorithms. This approach was locally evaluated considering real-time health-related data from patient health profiles, and the approval task was performed by a cloud service [95]. One method based on an encryption approach known as radio-frequency recognition is also used to provide security for medical data [96]. The flow of data in a network environment is quite complex for health information, and data security relies on biometric security with resource limitations in wearable health tracking systems.

Therefore, IoT is integrated in medical devices in which information is processed to optimize security for various medical applications. The service provided by the cloud consists of three servers, i.e., a database server, key-assigning center and authentication server. For healthcare data, secure cryptography is used based on a lattice framework. This system consists of four stages, i.e., data encryption, data decryption, key generation and a setup stage; a lattice polynomial is used as input for the first two stages, and key assignment is implemented in form of the public and private keys. The second stage is shared by the data server, and in the final stage, the data are treated as an input parameter and integrated with a random polynomial. If a user wants to forward a request to deal with medical data, the key-assigning center sends the hidden key pair to the data server through a secure channel, and the data server executes the blank text, taking the input and hidden key pair.

Security has been deployed in low-energy Bluetooth in a variety of ways, the most secure of which is LE secure relation, which follows a numeric comparison approach, and the ECHO (elliptical curve Hellman—Diffie) algorithm, which involves the use of a private and public key for every device to achieve secure key sharing. Two keys in the form of a master and slave are used, i.e., a recognition-resolving key and a signature-resolving key [97]. Potential attackers can be prevented from using 128-bit advanced encryption standard. The attacker’s identity, eavesdropping and man-in-the-middle can be effectively tackled using the security feature of low-energy Bluetooth. For Zigbee, various security features are available, some of which are optional and are used by network developers.

The security modes of Zigbee are based on 128 advanced encryption standards with security keys, i.e., master, link and network keys. The optional master key is used to secure the sharing and creation of the link, whereas the network key is essential and is shared by all devices using a network layer security process that manages all transmissions inside the network. The link layer is used to secure transmission in the application layer [98]. In a recent study, it was found that by sniffing the link key, a network can be easily exploited during device joining. From a healthcare point of view, if Zigbee is to be deployed, it must follow all optional security processes and trustable key management protocols.

In Sigfox, security is deployed using a private key attached to each message to minimize the chances of intercepting and spoofing attacks. Advanced attackers target the service provider and hardware node and may disclose the unique key, affecting healthcare systems. Message limitations, i.e., limited to about 140 messages per day, minimize the effects of bluff attacks; in the case of healthcare, even a small amount of fraudulent information affects the performance of the healthcare system and can degrade the patient’s condition. The performance of Sigfox is suitable for non-critical purposes for which acknowledgements and message delivery receipts are unimportant. However, for healthcare applications, successful message transmission at an appropriate speed is essential. Security compromises may be harmful to patients and affect medical records. Therefore, it is recommended that Sigfox be avoided for critical healthcare applications.

The LoRaWAN standard provides security by assigning a unique key to every node in the network that is known only to the network provider and the node. This measure can prevent man-in-middle attacks, with percept encoded and not interpretable [99]. A node with a unique key would fail, and the key would be detected using sophisticated castrate hardware or using a network attack in the server. If a key were identified, then attackers could easily use future messages from the node or transmit malicious messages to the base station. For appropriate security, key management must be taken care of by service providers and developers to protect sensitive data in healthcare databases from malicious attacks. Therefore, LoRaWAN can not sufficiently provide total security.

Narrowband IoT follows 3 GPP S3 security levels both in the application and transport layers [100]. It consists of several mechanisms for device identification, entity verification, user identity, data integrity, etc. It also supports optional mechanisms to ensure data confidentiality, application authentication, etc. An optional encryption approach is recommended for real threats that arise in radio communication to protect delicate health-related data. The use of narrowband IoT with all optional and mandatory mechanisms is a secure approach for healthcare applications. It is secure and highly efficient and supports long-range communication between multiple devices.

In the application layer scheme, a signal-scrambling key is delivered to authorized parties. The algorithm should be distributed in small data packets to reduce the risk of attacks, as it contains statistically crucial characteristics that reduce the probability of successful transmission and make it easy for an attacker to access the data. The steganography-based technique is used to protect electronic health data using encryption techniques and conceal the data inside the ECG signal, preventing discovery of hidden messages within the health records [101]. The homography encryption method is also used to secure data, allowing for encryption using a public key before it is sent to the cloud. It also follows mathematical functions over the data being encrypted, which enables machine learning without decrypting information in the cloud. If the request is sent by an authorized user, the system using an authority key can use a secure channel with a secret key to encrypt the data. This technique is a promising methods to integrate machine learning and security, but it has not been implemented in IoT-based health care [102].

WBAN applications involve the use of public spaces such as exercise areas and parks. In all cases, users have IOT devices such as smartwatches that collect biosignals from sensors implanted in the human body. The biosignal received from the body is sent to the static sink in the deployed area. Sink nodes contain 5G modules to send the information to the healthcare center for the processing [103]. Sensor nodes face many operational problems because of their low computing capacity, and mobility represents an open challenge in wireless body area networks, which are affected by the loss of messages due to topological calculation. Herein, we discuss a advanced version of MAC named MT-MAC, which was designed to ensure successful delivery of messages. A node handover approach between virtual clusters was considered for network integration, and the concept of connected dominated sets was used to achieve efficient energy transfer [104]. Voice data can also be used to track a person suffering from a viral infection; such a technique is suitable in the case of COVID-19, in which social distancing is important. Such a dataset can be used in comparison with an original voice database to support healthcare management [105]. The use of a deep reinforcement teaching method based on intelligent routing for IoT in WSNs can significantly reduce latency and increase the life time of the network. This suggested algorithm separates the complete network into unequal clusters relying on the present data load within the current sensor node, preventing the immature death of any network during communication [106]. A hierarchical approach for sensor networks in which transmission is more secure with longer node lengths and the operation of the network is improved with shared important management policy relies on the LEACH protocol [107].

## 7. Analysis and Results

To analyze the performance of narrowband IoT for deployment in standalone and in-band systems, we considered a bandwidth of 150 KHz for a general LTE cell. The framework consists of a tri-sector plot with a distance of 1600 m. Narrowband IoT for in-band communication is placed in the LTE system using a separate resource block. Narrowband IoT is placed in every cell using a similar resource block; the considered synchronized network and other considered parameters are shown in Table 3 for simulation.

The 150 KHz bandwidth consists of 10 subcarriers with 15 KHz spacing among uplink and downlink; compared to a single tone in uplink, this configuration achieves worse performance, i.e., 15 KHz or 3.74 KHz, and the achieved result in our case provides a lower jump compared to a single-tone IoT-based system. Therefore, we found it more practical to analyze narrowband IoT behavior.

The rate of the data (${RD}_{j,n}$) for $n^{th}$ resource block and node j is:

(1) $${RD}_{j,n}=Bandwidth(1+SIN.{RD}_{j,n})$$

where SIN is the signal to noise plus interference and is expressed as:

(2) $$SIN.{RD}_{j,n}=\frac{T_{p}C_{j,n}}{{Inter}_{j,n}+N(0)}$$

where $T_{p}$ denotes the transmitted power, $C_{j,n}$ is the gain of channel between the base station and node ‘j’ of the resource block, ${Inter}_{j,n}$ is the interference noticed by ‘j’ and considered to be minimum and $N\left(0\right)$ is power spectrum of noise volume. Depending on the SIN, the comparable coupling loss is calculated as:

Marked SIN = 164 + Power transmitted − Noise Margin − Bandwidth 10 log_10_ − coupling loss (3)

For analysis purposes, a combined chase approach is used, which relies on coupling loss so that similar information is renewed ‘N’ number of times. The renewed values for different coupling loss values are shown in Table 4.

The incoming duration is scattered in three stages of successive transmissions with equal incoming durations of 10 min, 40 min and 2 h, corresponding to 44.6%, 30.4% and 30.3% of devices, respectively. The mean appearance record per device is evaluated as:

(4) $$\frac{0.446}{300}+\frac{0.304}{1800}+\frac{0.303}{3600}=1.73\times 10^{-3}\frac{packets}{gadgets}(sec)$$

The network rate (NR) for packets relative to the quantity of devices is evaluated as:

(5) $$\frac{NR(packet)/cell}{1.73\times 10^{-3}\frac{packets}{gadgets}}=712\times NRgadget/cell$$

The general sensor types, along with their size, data coverage and duration of transmission, are shown in Table 5.

**Figure 5:**
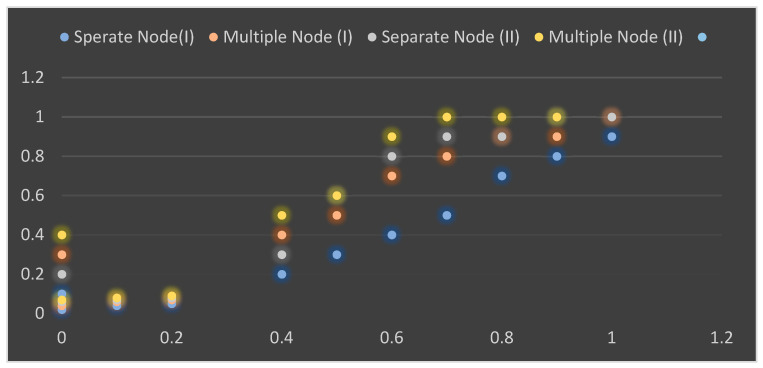
Average throughput for various implementation scenarios for separate nodes and multiple nodes. Separate node (I), in-band; separate node (II), standalone; multiple nodes (I), in-band; multiple nodes (II), standalone.

The performance of the system level for narrowband IoT is analyzed using the Matlab tool for supporting devices, effective throughputs and delay. Considering the parameters mentioned in Table 3 and Table 4, a simulation was conducted for both standalone and in-band communication for 600 random samples. For different implementation scenarios such as separate node patterns and multiple-node patterns, the cumulative distribution function evaluates the successful means in terms of throughput, which is the quantity of information transfer bits per second relative to the total overhead of authorized information, as shown in Figure 5.

Figure 5 shows that the obtained successful throughput is significantly smaller than the maximal data cost of 232.4 kbps and 250 kbps at the time of downlink and uplink, respectively, for the proposed narrowband IoT in the literature. This situation arises because of the shorter information of packet length compared to the overhead of the regulated channels, especially the physical control downlink, which requires a large number of repetitions for proper transmission. In the case of uplink, 40% of the allocated resources are protected for physical narrowband channel access (random), and some are protected for narrowband physical (shared channel) patterns with two packets, which degrades the result, whereas the use of multiple nodes improves the packet size and increases the gain in term of throughput.

**Figure 6:**
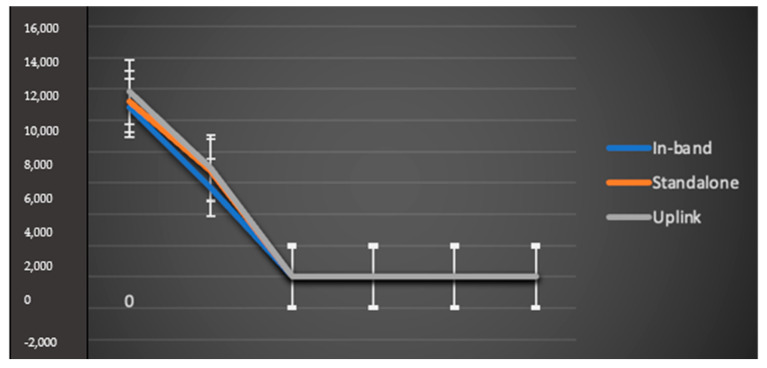
Patients served per cell (*X*-axis) for both separate-node and in-band configurations.

**Figure 7:**
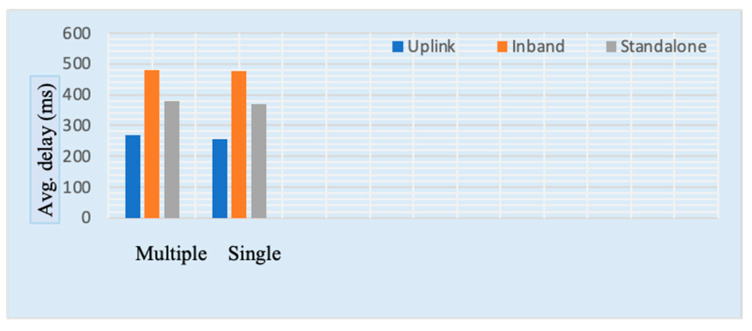
Average delay for single-node and multiple-node configurations.

In the case of in-band implementation, performance is degraded significantly compared to a standalone configuration because of LTE regulation. Figure 6 shows the average quantity of patients that can be included both for multiple and separate nodes in different deployment cases. It was also observed that multiple nodes increase the number of supported patients. This result shows that an increase in packet size minimizes the quantity of information that can be sent per patient, resulting in a lower overhead rate so that more patients can be monitored. Figure 5 and Figure 6 show that with an increase in the size of packets, the constructive throughput also increases. However, such an increase may cause a longer delay, as shown in Figure 7.

Figure 7 represents the delay for narrowband IoT under different conditions, where delay is the average duration required to finish the transfer with the desired overhead regulated information. It was also observed that with increased packet size, the time required for transmission also increases, resulting in increased delay for every device in the network. However, an increment in packet size improves the overall throughput, enabling more patients per cell to be served. Therefore, it is very clear that the quantity of supported devices, packet size, delay and throughput rely on one another.

The communication protocols considered in the analysis are the message queuing telemetry transport protocol (MQTPP) [108] and the limited application protocol (LAP), for which the mean transfer duration of the central server was calculated as [109]:

(6) $$T_{duration}=\frac{ST_{n}-ST_{1}}{Complete\;packets}$$

where *ST_n_* and *ST_1_* denote the time stamp of end packets and initial packets, respectively. The above communication protocol was considered to identify the efficiency of minimizing the time delay (average). Analysis of the central server data packets of different sizes is depicted in Table 6.

The total packet loss was evaluated using the following equation [108]:

(7) $$Rate\;of\;packet\;loss\left(\%\right)=(1-\frac{Sucessfull\;transmission}{Compete\;packet})$$

**Figure 8:**
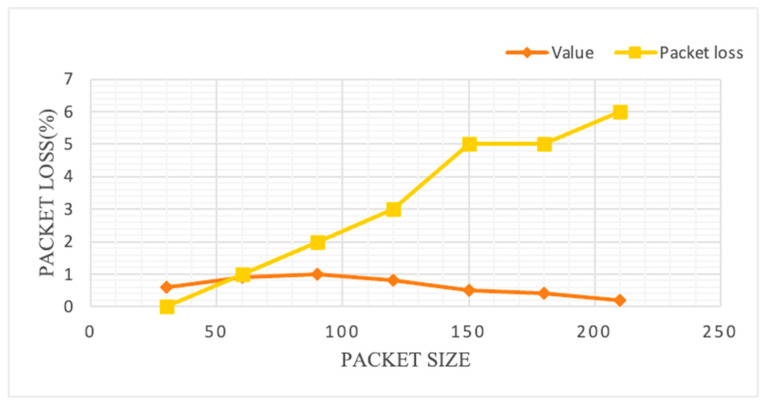
Packet size vs. packet loss (MQTTP).

The packet loss with respect the packet size is shown in Figure 8 for MQTTP, which shows that with increasing packet size, the packet loss also increases. Therefore, it is necessary to continue tracking the size of packet while sending the information in the network to maintain the quality with minimal loss.

**Figure 9:**
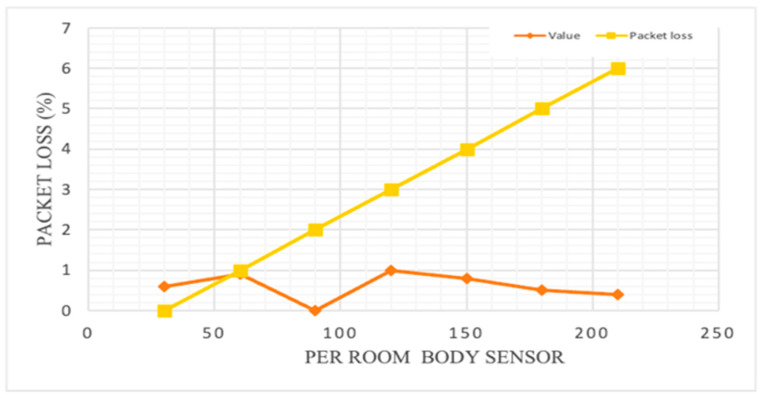
Sensor nodes vs. packet loss per room (MQTTP).

**Figure 10:**
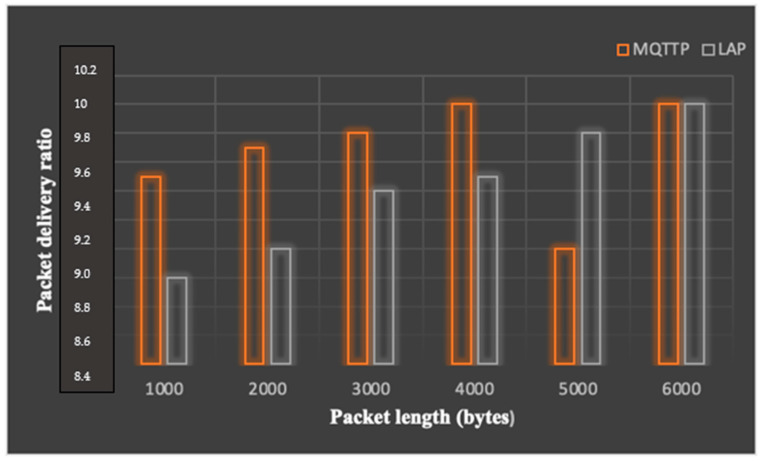
Throughput of MQTTP and LAP.

Keeping the above parameters fixed, the network was simulated by changing the sensor quantity per room and the packet length. The packet delivery ratio is shown in Figure 9, which is expressed as:

(8) $$Packet\;delivery\;ration=\frac{z}{\sum_{i=1}^{N}y_{i}}$$

where *y_i_* denotes the total packets, ‘*i*’ is the node and *N* is the total nodes in a network. The throughput of the network for MQTTP and LAP is depicted in Figure 10.

## 8. Conclusions

The proposed model can be implemented in the future based on IoT for healthcare systems. This system may be suitable for observations in specific situations, as well as for implementation in general system. Various unnoticeable, wearable sensors were discussed and analyzed, with a primary focus on healthcare applications. Long- and short-range communication standards were also compared to reflect the suitability of this approach for healthcare applications. We observed that IoT (narrow band) and Bluetooth low energy (BLE) are the most acceptable modes for long- and short-range communication with respect to healthcare applications. Cloud computing was found to be best means of handling and organizing large amounts of data. It was also observed that encryption and acquire command policies greatly enhanced safety; however, for real-time applications, no such standard is available for wearable healthcare systems relying on IoT.

With the modernization of wearable technologies in various areas, as well as cloud and communication technologies, we identified various significant domains for upcoming future research. Encryption methods based on lightweight storage in the cloud and machine learning present opportunities for researchers to explore build new approaches in the domain of healthcare system relying on IoT. The vital objective of our paper was to identify the activities and issues associated with the field of IoT for healthcare system applications. Existing evidence shows that IoT greatly supports patient monitoring and data to delivery to control centers. ECG tracking systems can be implemented using machine learning approaches to easily identify any disease. Researchers have applied various approaches to reduce consumption by implementing algorithms to improve efficiency. An IoT system with existing features can reliably, effectively and adaptively track patient condition, as well as the health of elderly patients, using specific sensors, cameras, speakers. etc.

In the future, IoT systems can be improved by using a suitable power absorption model with a secure cloud platform to enhance security performance. We conducted a performance analysis of narrowband IoT using single- and multi-node approaches for healthcare applications and found that multiple-node deployment crucially performs well in term of the quantity of patients and throughput with an increase in delay. Therefore, it is important to optimize the desired delay, throughput and device density. Reliability and efficiency were analyzed with the help of two protocols; we found that for small packets, the message queuing telemetry transport protocol (MQTTP) was efficient compared to limited application protocol (LAP) in sending information from the sensor architecture to the central server. MQTTP also performs well in high-traffic scenarios with a small packet size. Therefore, MQTTP may be considered an efficient protocol for communication in smart healthcare systems. Scheduling mechanisms can help to improve the overall communication performance to deliver packets with appropriate analysis of transmission in the future.

## Figures and Tables

**Table 1:** Comparison of long-distance communication standards.

Parameters	IoT (NB)	Big Fox	LoRa WAN
Operating Band	In-band LTE mode, guard-band LTE	915 MHz for US and 868 MHz for Europe	915 MHz for US and 868 MHz for Europe
License required	Yes	No	No
Approx. range	16 km	10 km	7 km
Rate of data	250 kbps	120 kbps	Up to 6 kbps
Capacity of network	54,000 nodes	55,000 nodes	42,000 nodes
Relevance for health care	Good	Poor	Average
Direction of communication	Downlink and uplink	Request for downlink and uplink facility is unlimited	Downlink and uplink
Safety attributes	Security enabled with S3 (3G PP), which includes authentication, recognition and confidentiality with data integrity	A private key is used with a limited of 142 messages with scrambling and encryption enabled	Each node is assigned a unique key in the network, which is known to the network and supports data encryption

**Table 2:** Comparison of short-distance communication standards.

Parameter	Zigbee	Bluetooth
Operating band	2.5 GHz	2400–2484 MHz
Topography	Mesh topology	Star topology
Radius	10–100 m	30 feet
Rate of data	250 kbps	24 Mbps
Safety attribute	A network with an encryption key (128-AES) and a link key is available to provide additional security in the application layer	Pairing is implemented in the secure mode to exchange information and provide two-key authentication and protection
Relevance for healthcare	Average	Better

**Table 3:** Parameters for simulation.

Parameters	Values
Distance (interarea)	1600 m
Frequency	800 MHz
Cell design	Grid (hexagonal)
Transmission power	28 dB (per 200 KHz)
Model for path loss	I = 110.8 for 800 MHz
User tools (noise figure)	2 dB
Base station (noise figure)	4 dB
Cell–area correlation	0.6
Cell–location correlation	1.0
Standard deviation	6 dB
Spectral volume	(−164 dBm/Hz)
Antenna gain	16 dBi

**Table 4:** Repetition element (category-wise range).

Coupling Loss	Renewed
Under 140 dB	One (01)
141–143 dB	Two (02)
144–146 dB	Four (04)
147–149 dB	Eight (08)
150–152 dB	Sixteen (16)
153–155 dB	Thirty-two (32)
156–158 dB	Sixty-four (64)
159–161 dB	One hundred twenty-eight (128)
Above 162 dB	256 used for downlink and 128 for uplink

**Table 5:** Sensor types and their corresponding data coverage, length of information and period.

Types of Sensor	Data Coverage	Size of Information	Period
Motion	---------	Two bytes	Two hours
Hear Beat	0–60 beats/min	One byte	Four to five minutes
Blood Pressure	20–350 mm Hg	Two bytes	Half an hour
Temperature (Body)	23–45 °C	One byte	Four to five minutes
Rate (Respiratory)	3–50 breaths per min	One byte	Four to five minutes
pH (Blood)	6.6–7.6 units	One byte	Four to five minutes

**Table 6:** Central server data packet analysis.

Packet Size	*τ* ± *ρ* (LAP)	*τ* ± *ρ* (MQTPP)
30	2.428 ± 0.001	0.091 ± 0.004
60	5.332 ± 0.002	0.764 ± 0.006
600	6.712 ± 0.006	2.128 ± 0.016

## Data Availability

Not applicable.
